# Supplementary material for: Retrospective Observational Study of CSF-Derived HIV-1 Tat and Vpr Amino Acid Sequences in a South African Pediatric Cohort with HIV Subtype C
Source: Int J Mol Sci. 2025 May 22;26(11):5008. doi: 10.3390/ijms26115008 (PMC12155176; doi:10.3390/ijms26115008)
Supplement: Supplementary file 1 [file ijms-26-05008-s001.zip › ijms-3642972-supplementary.pdf]

Table S1: Study characteristics of study population.

Participant no.	Age (in months)	Sex	CD4 count (cells/µL)	Viral load (copies/mL)	Treatment status
C-SA_1	78	Male	5,34	49 000	Untreated
C-SA_2	11	Male	16,95	N/A	Untreated
C-SA_3	39	Female	270	N/A	Untreated
C-SA_4	7	Male	533	N/A	ART

Table S2: Tat and Vpr protein sequences for study participants.

**Sample ID**	**Accession number**	**Tat sequence**
Tat-C-SA_1	PP437871	MEPVDPNLEPWNHPGSQPKTPCN**K**CYCK**R**C**S**YHCLVCFQTKGLGISYGR KKRRQRR**S**XXPSS**E**DHQNPISKQ
Tat-C-SA_2	PP437872	MXPVDPNLEPWNHPGSQPKTACN**T**CYCK**H**C**S**YHCLVCFQTKGLGISYGR KKRRQRR**R**APPSS**E**DHQNLISKQ
Tat-C-SA_3	PP437873	MEPVDPNLEPWNHPGSQPITPCN**S**CYCK**X**C**S**YHCLVCFQKKGLGISYGR KKRRQRR**S**APPXSXDHQNLXSKQ
Tat-C-SA_4	PP437874	MEPIDPNLEPWNHPGSQPKTPCT**K**CFCK**A**C**S**YHCLVCFQKKGLGISYGR KKQRQRR**S**APPSSGDHQNPVSKQ
**Sample ID**	**Accession number**	**Vpr sequence**
Vpr-C-SA_1	PP437875	MEQAPEDQGPQREPYNEWVL**E**XLEELKQEAVRXFPRPWLH**X**LGQ**Y**XYE TYGDTW**T**GXEVIXRIXQQLLFIHFRIGC**X**HSRIGILXQRRARXGASRP
Vpr-C-SA_3	PP437876	MEQAPEDQGPQREPYNEWAL**E**ILEELKQEAVRHFXRPWLQ**S**LGQ**Y**IYDT YGDTW**T**GVESIMRIXQRMLFIHFRMGC**Q**HSRIGIMRQRRXRNGASRS
Vpr-C-SA_4	PP437877	MEQAPEDQGPQREPYNEWAL**X**LLEELKQEAVRHFPRXWLH**G**LGQ**H**IYE THGDTW**E**GVEALIRFLQQLXFTHFRIGC**Q**HSRIGIVRQRRARNGANRS

The highlighted and underlined represent amino acids/positions that have previously been linked to HIV-1 neuropathogenesis. Amino acid positions with ambiguous base calls, due to mixed nucleotide signals in Sanger sequencing chromatograms, are denoted by "X". These ambiguities likely reflect underlying viral quasispecies diversity within the CSF samples. No premature stop codons were detected in the open reading frames.

Table S3: Differences in the frequency of Tat amino acids between CSF-derived sequences from our cohort and blood-derived sequences from the Los Alamos database

Residue variant	Tat-C-SA_s<br>N = 4	Tat-C-SA_EU<br>N = 5	p-value	FDR
**L3P**<br>**P3L**	4 (100%)<br>0	4 (80%)<br>1 (20%)	0.343	0.500
**K7N**<br>**N7K**	4 (100%)<br>0	4 (80%)<br>1 (20%)	0.343	0.500
**K12N**<br>**N12K**	4 (100%)<br>0	4 (80%)<br>1 (20%)	0.343	0.500
**T24K**<br>**K24T**<br>**K24S**<br>**K24N**	2 (50%)<br>1 (25%)<br>1 (25%)<br>0	3 (60%)<br>1 (20%)<br>0<br>1 (20%)	0.549	0.607
**R29K**<br>**K29R**<br>**K29H**<br>**K29S**<br>**K29A**	0<br>1 (25%)<br>1 (25%)<br>0<br>1 (25%)	2 (40%)<br>1 (20%)<br>1 (20%)<br>1 (20%)<br>0	0.422	0.554
**C31S**<br>**S31C**	4 (100%)<br>0	3 (60%)<br>2 (40%)	0.151	0.500
**Q35L**<br>**L35Q**	4 (100%)<br>0	3 (60%)<br>2 (40%)	0.151	0.500
**L39Q**<br>**Q39L**	4 (100%)<br>0	3 (60%)<br>2 (40%)	0.151	0.500
**R57S**<br>**S57R**<br>**S57N**	3 (75%)<br>1 (25%)<br>0	4 (80%)<br>0<br>1 (20%)	0.358	0.500
**S59P**<br>**P59S**	3 (75%)<br>0	4 (80%)<br>1 (20%)	0.358	0.500

Table S4: Differences in the frequency of Vpr amino acids between CSF-derived sequences from our cohort and blood-derived sequences from the Los Alamos database

Residue Variant	Vpr-C-SA_s<br>N = 3	Vpr-C-SA_EU<br>N = 5	p-value	FDR
**A4P**<br>**P4A**	0<br>3 (100%)	5 (100%)<br>0	**0.005**	0.125
**Y15H**<br>**H15Y**	0<br>3 (100%)	1 (20%)<br>4 (80%)	0.408	0.51
**V19T**<br>**T19V**<br>**T19A**	0<br>1 (33%)<br>2 (66%)	2 (40%)<br>0<br>1 (20%)	0.161	0.420
**E21I**	0	0	-	-
**L22V**<br>**V22L**<br>**V22I**	0<br>1 (33%)<br>1 (33%)	3 (60%)<br>1 (20%)<br>1 (20%)	0.292	0.487
**D25E**<br>**E25D**	3 (100%)<br>0	4 (80%)<br>1 (20%)	0.408	0.510
**G41S**<br>**G41D**<br>**S41G**	1 (33%)<br>0<br>1 (33%)	4 (80%)<br>1 (20%)<br>0	0.205	0.427
**I42L**<br>**L42I**	3 (100%)<br>0	4 (80%)<br>1 (20%)	0.408	0.510
**H45Y**<br>**Y45H**	2 (66%)<br>1 (33%)	4 (80%)<br>1 (20%)	0.673	0.717
**T55A**<br>**A55T**<br>**T55I**<br>**T55E**	0<br>2 (66%)<br>0<br>1 (33%)	2 (40%)<br>2 (40%)<br>1 (20%)<br>0	0.292	0.487
**E58Q**<br>**Q58E**	3 (100%)<br>0	4 (80%)<br>1 (20%)	0.408	0.510
**T61I**<br>**I61T**<br>**I61M**	1 (33%)<br>0<br>1 (33%)	4 (80%)<br>1 (20%)<br>0	0.205	0.427
**M74I**<br>**I74M**<br>**I74L**	2 (66%)<br>1 (33%)<br>0	4 (80%)<br>0<br>1 (20%)	0.315	0.492
**H77Q**<br>**Q77H**<br>**Q77A**	2 (66%)<br>0<br>0	2 (40%)<br>2 (40%)<br>1 (20%)	0.292	0.487
**N94S**<br>**S94N**<br>**S94G**	2 (66%)<br>1 (33%)<br>0	3 (60%)<br>1 (20%)<br>1 (20%)	0.688	0.717

Table S5: Frequency of residue variants between Tat sequences from the United States of America, South Africa and India.

Residue variant	South Africa (Tat-C-SA_s) N = 4	South Africa (Tat-C-SA_EU) N = 5	India (Tat-C-IN_KF) N = 27	USA (Tat-B-USA_MK, M, U, and AYB). N = 10	p-value	FDR
**D2E**	3 (75%)	5 (100%)	27 (100%)	5 (50%)	< 0.001	**< 0.001**
**E2D**	0	0	0	4 (40%)		
**N12K**	0	1 (20%)	4 (15%)	10 (100%)	< 0.001	**< 0.001**
**K12N**	4 (100%)	4 (80%)	23 (85%)	0		
**T23N**	3 (75%)	4 (80%)	25 (93%)	1 (10%)	< 0.001	**< 0.001**
**N23T**	1 (25%)	1 (20%)	2 (7%)	8 (80%)		
**K24N**	0	1 (20%)	24 (89%)	4 (40%)	< 0.001	**< 0.001**
**N24K**	2 (50%)	3 (60%)	1 (4%)	1 (10%)		
**N24T**	1 (25%)	1 (20%)	0	3 (30%)		
**R29K**	0	2 (40%)	12 (44%)	7 (70%)	0.086	0.097
**K29R**	1 (25%)	1 (20%)	10 (37%)	1 (10%)		
**K29A**	1 (25%)	0	0	1 (10%)		
**K29H**	1 (25%)	1 (20%)	1 (4%)	0		
**K29S**	0	1 (11%)	0	1 (10%)		
**S31C**	0	2 (40%)	0	10 (100%)	< 0.001	**< 0.001**
**C31S**	4 (100%)	3 (60%)	27 (100%)	0		
**Y32F**	0	0	1 (4%)	8 (80%)	< 0.001	**< 0.001**
**F32Y**	4 (100%)	5 (100%)	26 (97%)	0		
**S57R**	1 (25%)	0	6 (22%)	9 (90%)	< 0.001	**< 0.001**
**R57S**	3 (75%)	4 (80%)	21 (78%)	0		
**S61D**	0	0	0	9 (90%)	< 0.001	**< 0.001**
**D61S**	4 (100%)	5 (100%)	27 (100%)	0		
**Q63E**	2 (50%)	5 (100%)	25 (93%)	1 (10%)	< 0.001	**< 0.001**
**E63Q**	0	0	0	6 (60%)		
**E63K**	0	0	2 (7%)	1 (10%)		
**T64D**	4 (100%)	3 (60%)	27 (100%)	0	< 0.001	**< 0.001**

**D64T**	0	0	0	4 (40%)		
**D64A**	0	1 (20%)	0	2 (20%)		
**D64I**	0	0	0	3 (30%)		
**L69I**	3 (75%)	4 (80%)	23 (85%)	0	< 0.001	**< 0.001**
**I69L**	0	0	0	10 (100%)		
**I69V**	1 (25%)	1 (20%)	5 (19%)	0		

Significant p–values were highlighted.

Table S6: Frequency of residue variants between Vpr sequences from the USA, Portugal, and South Africa.

**Residue variant**	**South Africa (Vpr-C-SA_s) N = 3**	**South Africa (Vpr-C-SA_EU) N = 5**	**USA (Vpr-B-USA_MK, M, and AF) N = 8**	**Portugal (Vpr-B-PR_KM) N = 1**	**p-value**	**FDR**
**A4P**	0	5 (100%)	0	0	0.007	**0.030**
**P4A**	3 (100%)	0	8 (100%)	1 (100%)		
**A19T**	0	3 (60%)	7 (88%)	1 (100%)	0.167	0.241
**T19A**	2 (66%)	1 (20%)	1 (12%)	0		
**I21E**	2 (66%)	5 (100%)	8 (100%)	1 (100%)	-	-
**I22L**	1 (33%)	1 (20%)	8 (100%)	1 (100%)	0.034	0.073
**L22I**	1 (33%)	1 (20%)	0	0		
**L22V**	0	3 (60%)	0	0		
**I24E**	3 (100%)	5 (100%)	8 (100%)	1 (100%)	-	-
**Q28N**	0	0	7 (88%)	0	< 0.001	**0.001**
**N28Q**	3 (100%)	5 (100%)	0	0		
**I37P**	2 (66%)	5 (100%)	0	0	0.046	0.086
**P37I**	0	0	4 (50%)	0		
**P37V**	0	0	3 (38%)	1 (100%)		
**G41S**	1 (33%)	4 (80%)	0	1 (100%)	0.054	0.089
**S41G**	1 (33%)	0	5 (63%)	0		

**G41N**	0	0	3 (38%)	0		
**H45Y**<br>**Y45H**	3 (100%)<br>1 (33%)	4 (80%)<br>1 (20%)	1 (13%)<br>7 (88%)	0<br>1 (100%)	0.012	**0.039**
**E48D**<br>**D48E**	1 (33%)<br>2 (66%)	2 (40%)<br>3 (60%)	1 (13%)<br>6 (75%)	0<br>1 (100%)	0.005	**0.030**
**T55A**<br>**A55T**	0<br>2 (66%)	2 (40%)<br>2 (40%)	5 (63%)<br>2 (25%)	1 (100%)<br>0	0.361	0.384
**Q77R**<br>**R77Q**	0<br>2 (66%)	0<br>2 (40%)	3 (38%)<br>5 (63%)	0<br>1 (100%)	0.197	0.256
**L84T**<br>**T84L**	0<br>1 (33%)	0<br>5 (100%)	6 (75%)<br>1 (13%)	0<br>0	0.024	0.061
**P85R**<br>**R85P**	2 (66%)<br>0	4 (80%)<br>1 (20%)	4 (50%)<br>3 (38%)	0<br>1 (100%)	0.385	0.385
**Q86R**<br>**R86Q**	0<br>3 (100%)	0<br>5 (100%)	2 (25%)<br>6 (75%)	1 (100%)<br>0	0.251	0.297

Significant p–values were highlighted.

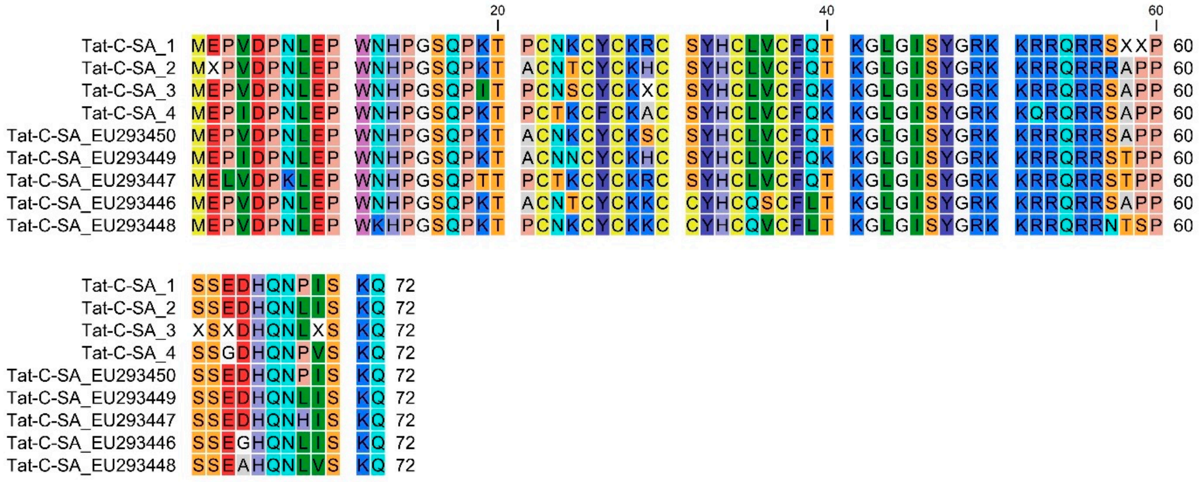

Figure S1: Multiple sequence alignment of CSF-derived subtype C Tat sequences with PBMC-derived subtype C Tat sequences originating from South Africa. Amino acid positions with ambiguous base calls, due to mixed nucleotide signals in Sanger sequencing chromatograms, are denoted by "X".

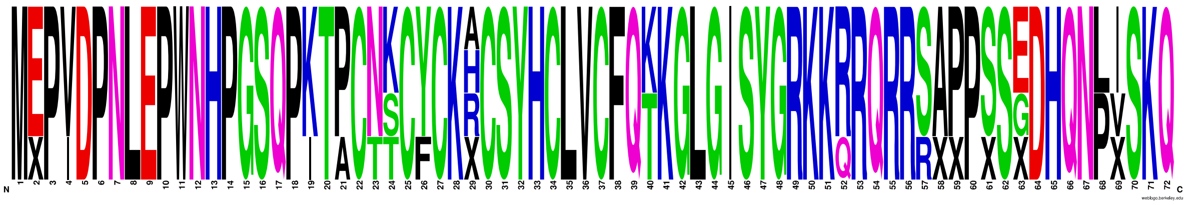

Figure S2: WebLogo showing the frequency of specific Tat amino acids at each position. The size of each amino acid's single-letter code reflects its frequency among CSF-derived and PBMC-derived subtype C Tat sequences originating from South Africa.

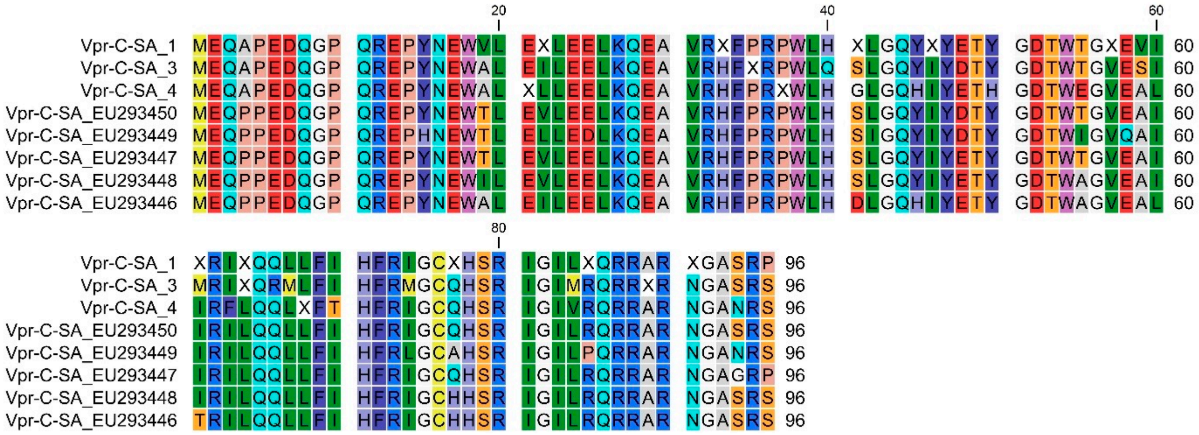

Figure S3: Multiple sequence alignment of CSF-derived subtype C Vpr sequences with PBMC-derived subtype C Vpr sequences originating from South Africa. Amino acid positions with ambiguous base calls, due to mixed nucleotide signals in Sanger sequencing chromatograms, are denoted by "X".

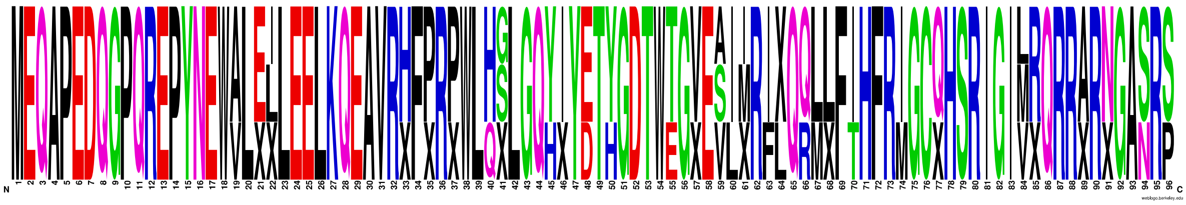

Figure S4: WebLogo showing the frequency of specific Vpr amino acids at each position. The size of each amino acid's single-letter code reflects its frequency among CSF-derived and PBMC-derived subtype C Tat sequences originating from South Africa.

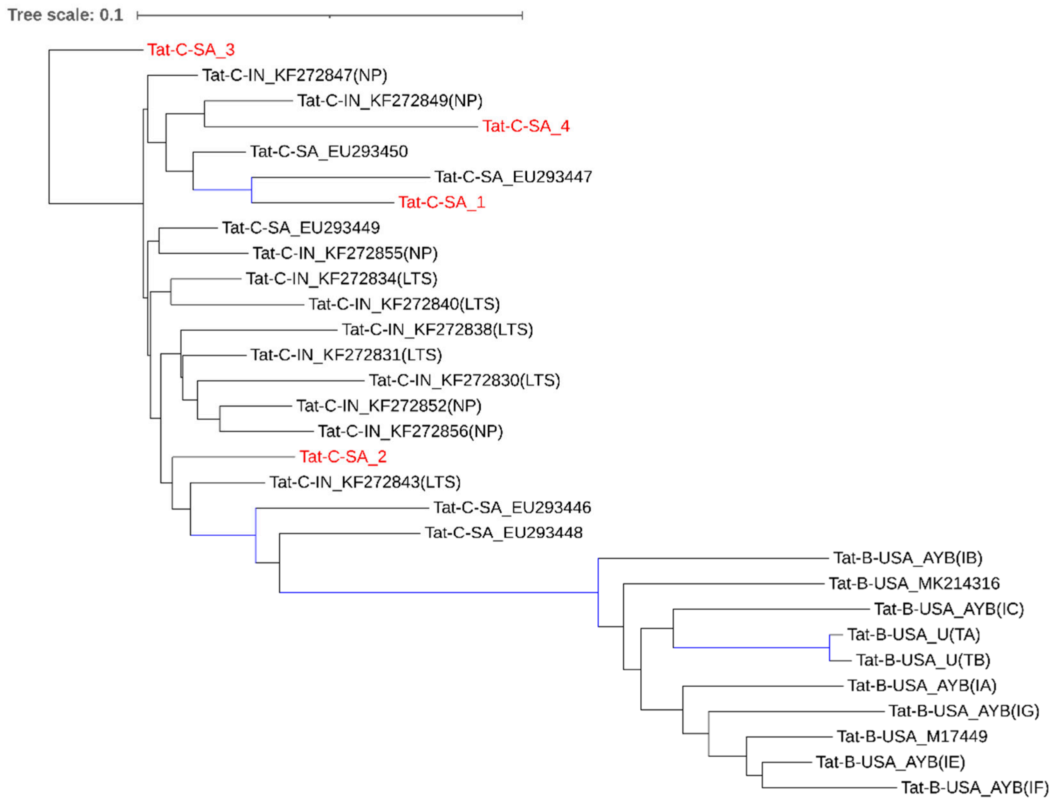

Figure S5: The maximum likelihood phylogenetic tree analysis comparing representative sequences of CSF derived subtype C Tat (red) with PBMC-derived Tat sequences originating from South Africa, India, and America constructed using the Kimura 80 model with gamma rate variation and topology variation (K80+G+T). The bootstrap probability (> 70%, 100 replicates) are highlighted in blue.
